# Supplementary material for: Synaptic Alterations Are Preceding the Axonal Loss in Optic Atrophy of Wolfram Syndrome Mouse Model
Source: bioRxiv. 2026 Mar 25:2026.03.22.713521. Preprint. [Version 1] doi: 10.64898/2026.03.22.713521 (PMC13041949; doi:10.64898/2026.03.22.713521)
Supplement: 1 [file NIHPP2026.03.22.713521V1-supplement-1.pdf]

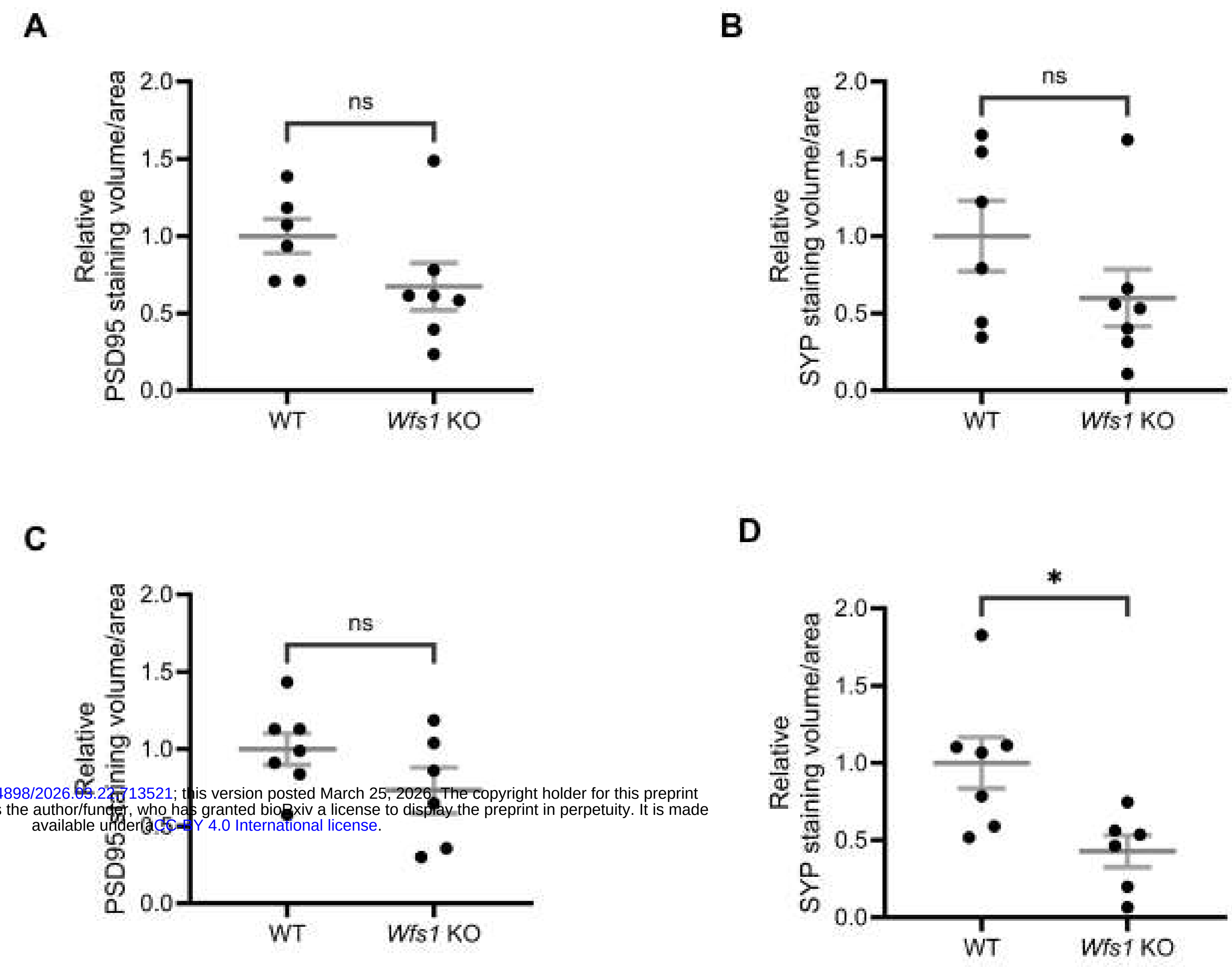

bioRxiv preprint doi: https://doi.org/10.64898/2026.03.23.713521; this version posted March 25, 2026. The copyright holder for this preprint (which was not certified by peer review) is the author/funder, who has granted bioRxiv a license to display the preprint in perpetuity. It is made available under aCC-BY 4.0 International license.

Supplementary Figure 1. Quantification of synaptic proteins in *Wfs1* KO mice.
Relative quantification of (A,C) PSD95 and (B,D) synaptophysin staining volume in 4 (A and B) and 7 (C and D) month old *Wfs1* KO mice compared to WT mice. Data are presented as normalized values ± SEM relative to WT. Statistical comparisons were performed using a two-tailed unpaired Student's t-test with Welch's correction. n ≥ 6. *P < 0.05. ns - not significant.

bioRxiv preprint doi: https://doi.org/10.64898/2026.03.22.713521; this version posted March 25, 2026. The copyright holder for this preprint (which was not certified by peer review) is the author/funder, who has granted bioRxiv a license to display the preprint in perpetuity. It is made available under aCC-BY 4.0 International license.

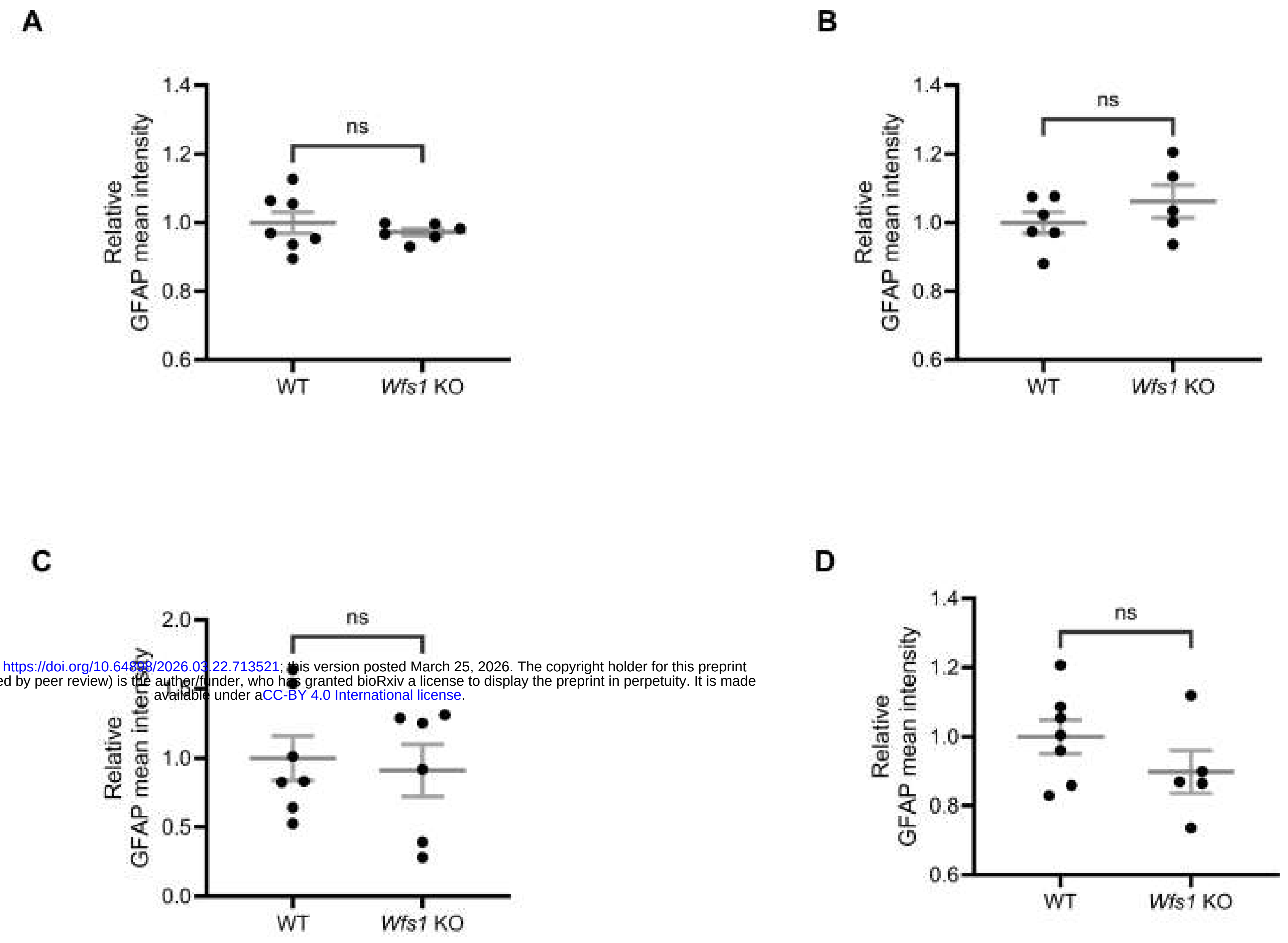

Supplementary Figure 2. Quantification gliosis state in retina and optic nerve of *Wfs1*-KO mouse model.
Relative quantification of GFAP staining mean intensity in retina (A and B) and optic nerve (C and D) of 4 (A and C) and 7 (B and D) month old *Wfs1* KO mice compared to WT mice. Values represent normalized means ± SEM relative to WT. Statistical comparisons were performed using a two-tailed unpaired Student's t-test with Welch's correction. n ≥ 5. ns - not significant.

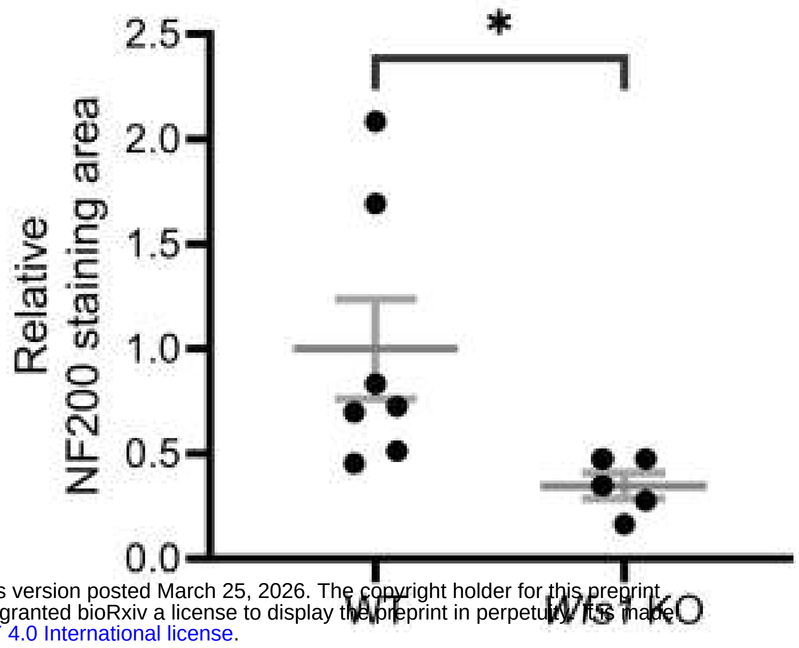

bioRxiv preprint doi: https://doi.org/10.64898/2026.03.22.713521; this version posted March 25, 2026. The copyright holder for this preprint
(which was not certified by peer review) is the author/funder, who has granted bioRxiv a license to display the preprint in perpetuity. It is made
available under aCC-BY 4.0 International license.

Supplementary Figure 3. Quantification of NF200 staining area in optic nerve section of *Wfs1* KO model.
Relative quantification of NF200 staining area in optic nerve sections of 7 month old *Wfs1* KO mice compared to WT mice. Data points represent normalized values ± SEM relative to WT. Statistical comparisons were performed using a two-tailed unpaired Student's t-test with Welch's correction. n ≥ 5. *P<0.05.
